# Coupled Enrichment of Cu and Sn at the Oxide/Steel Interface and Its Regulation by Si in Recycled Steels

**DOI:** 10.3390/ma19112370

**Published:** 2026-06-02

**Authors:** Jiahao Qiang, Fangbo Yang, Yuhe Huang, Jun Lu, Shuize Wang, Xinping Mao

**Affiliations:** 1 Institute for Carbon Neutrality, University of Science and Technology Beijing, Beijing 100083, China; 18500713877@139.com (J.Q.); yfb12138@163.com (F.Y.); lujun@ustb.edu.cn (J.L.); maoxinping@126.com (X.M.); 2 Institute for Steel Sustainable Technology, Liaoning Academy of Materials, Shenyang 110004, China

**Keywords:** Cu-Sn coupled enrichment, interfacial segregation, high-temperature oxidation, Si regulation, recycled steels

## Abstract

The accumulation of residual elements such as Cu and Sn in recycled steels has become an increasingly critical issue, as their enrichment during high-temperature oxidation can lead to surface hot shortness and deterioration of surface quality. In this work, the coupled enrichment behavior of Cu and Sn at the oxide/steel interface and its regulation by Si were systematically investigated through high-temperature oxidation experiments and microstructural characterization. The results reveal that selective oxidation of Fe during high-temperature exposure leads to the rejection of Cu toward the oxide/steel interface, resulting in significant interfacial enrichment. The presence of Sn further intensifies this enrichment by lowering the melting point of the Cu-rich phase and promoting the formation of Cu–Sn liquid films along grain boundaries, thereby aggravating intergranular penetration and surface degradation. In contrast, the addition of Si effectively suppresses the interfacial enrichment of Cu and Sn. Microstructural analyses indicate that Si promotes internal oxidation and facilitates the formation of Si-containing oxides such as Fe_2_SiO_4_ within the oxide scale and near the interface, which modifies the interfacial structure and limits the diffusion and accumulation of Cu-rich phases. Consequently, the formation and penetration of Cu–Sn liquid are significantly inhibited. These findings clarify the coupling mechanism of Cu and Sn during oxidation and reveal an effective Si-based strategy for mitigating the detrimental enrichment of residual elements in recycled steels, providing guidance for improving the surface quality of steels produced from scrap-containing charges.

## 1. Introduction

The global steel industry is undergoing a rapid transition toward low-carbon and resource-efficient production. Increasing the utilization of recycled steel scrap has been widely recognized as an effective pathway to reduce energy consumption and CO_2_ emissions associated with primary ironmaking routes [1,2]. However, the repeated recycling of steel scrap inevitably leads to the accumulation of residual elements that cannot be effectively removed during conventional steelmaking processes [3]. Among these elements, copper (Cu) and tin (Sn) are considered two of the most typical tramp elements due to their high stability and limited removability during refining. As the proportion of recycled scrap increases in modern steel production, the influence of these residual elements on surface quality and hot workability has become an increasingly critical metallurgical issue [4].

During high-temperature oxidation and hot processing, residual elements may undergo selective redistribution due to differences in oxidation affinity and diffusion behavior. In particular, Cu is well known to accumulate at the oxide/steel interface because of its lower oxidation tendency compared with iron, leading to local enrichment beneath the oxide scale [5]. When the interfacial Cu concentration exceeds its solubility in austenite, a Cu-rich liquid phase may form within a critical temperature range, which can penetrate along grain boundaries and induce surface hot shortness during subsequent deformation [5,6]. Although this phenomenon has been extensively investigated, most previous studies mainly focused on the behavior of Cu alone and its influence on surface cracking during hot rolling.

In contrast, tin (Sn), another important tramp element originating from recycled scrap, has received comparatively less attention despite its increasing concentration in modern steels [5,7]. Sn exhibits a strong tendency to segregate at grain boundaries and metal/oxide interfaces during high-temperature exposure, and it may significantly modify oxidation kinetics, scale adhesion, and interfacial diffusion processes [8,9]. More importantly, recent studies suggest that Sn may interact with Cu during high-temperature oxidation, potentially influencing the enrichment behavior and stability of Cu-rich phases at the oxide/steel interface [10]. Such coupled enrichment of Cu and Sn may play a crucial role in determining the interfacial chemistry and the susceptibility of recycled steels to surface degradation [5]. However, compared with the well-established understanding of Cu-induced hot shortness, the synergistic redistribution and enrichment mechanisms of Cu and Sn during oxidation remain insufficiently clarified.

Alloying regulation has been considered an effective strategy to modify oxidation behavior and mitigate the detrimental effects of residual elements [11]. Silicon (Si) is known to strongly influence oxide scale formation by promoting internal oxidation or the formation of Si-rich sublayers near the oxide/steel interface [12]. These Si-containing oxide structures can alter the diffusion pathways of both oxygen and metallic elements, thereby potentially affecting the redistribution and enrichment of tramp elements during oxidation. Nevertheless, how Si affects the coupled enrichment behavior of Cu and Sn at the oxide/steel interface has not yet been systematically investigated.

Therefore, the present study aims to elucidate the coupled enrichment behavior of Cu and Sn at the oxide/steel interface in recycled steels and to explore the regulatory role of Si in modifying this process. Through controlled high-temperature oxidation experiments combined with detailed microstructural and compositional characterization, the evolution of interfacial enrichment, elemental redistribution, and oxide scale morphology were systematically analyzed. Particular attention is given to the interaction between Cu and Sn during oxidation and the influence of Si on the interfacial enrichment behavior. The results provide new insights into the synergistic effects of residual elements in recycled steels and offer potential alloy design strategies for mitigating surface degradation during high-temperature processing.

## 2. Materials and Methods

To investigate the coupled enrichment behavior of residual elements during high-temperature oxidation, a series of experimental steels containing typical tramp elements were designed. Particular attention was given to copper (Cu) and tin (Sn), which are two of the most common residual elements accumulated in recycled steels. In addition, silicon (Si) was introduced as a regulatory element to examine its influence on interfacial enrichment behavior. The experimental steels were produced using a vacuum induction furnace and cast into billets. The chemical compositions were determined by optical emission spectroscopy (OES), and the measured compositions are summarized in Table 1. The Cu and Sn content was maintained at approximately 0.3 wt.% and 0.05 wt.% respectively to simulate typical Cu and Sn levels in scrap-based steels, while Si content was adjusted to 0.4 wt.% to evaluate its influence on Cu-Sn interfacial enrichment behavior. After casting, the billets were homogenized at 1200 °C for 1 h in an argon atmosphere to eliminate chemical segregation. Subsequently, the billets were hot rolled in five passes to reduce the thickness from 40 mm to 3 mm, with a finishing rolling temperature of approximately 830 °C. After rolling, the sheets were laminar-cooled to 550 °C, followed by furnace cooling to room temperature.

**Figure 1:**
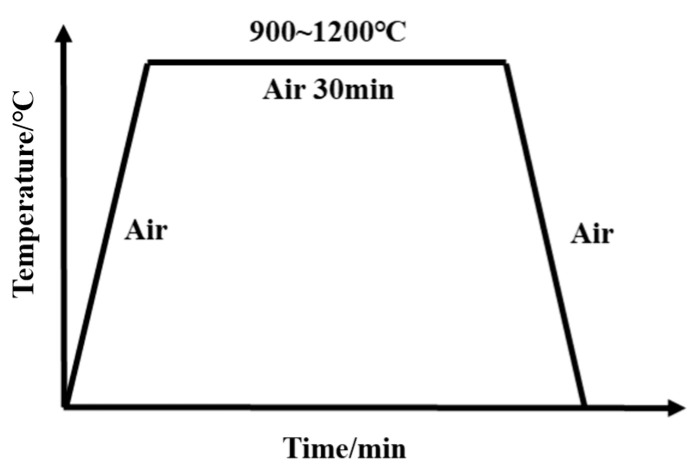
Schematic illustration of the thermal cycles used for high-temperature oxidation experiments.

Rectangular specimens (~10 mm × 10 mm × 3 mm) were sectioned from the hot-rolled sheets for oxidation experiments. Prior to oxidation, the specimens were mechanically ground using SiC papers from 400 to 2000 grit, followed by diamond polishing to obtain a mirror surface. The polished samples were ultrasonically cleaned in ethanol and dried with warm air. High-temperature oxidation experiments were conducted to simulate industrial thermal conditions during hot processing. The samples were heated in a resistance furnace under an argon atmosphere to the target temperatures, followed by exposure to laboratory air for 30 min to induce oxidation. After oxidation, the samples were cooled in air to room temperature. To evaluate oxidation kinetics, the oxidation-induced mass gain was measured using an analytical balance with an accuracy of 0.01 mg. The thermal cycles used in the oxidation experiments are schematically illustrated in Figure 1.

After oxidation, the samples were sectioned along the rolling direction using wire electrical discharge machining (EDM). Cross-sectional specimens were prepared through standard metallographic procedures, including mechanical grinding and diamond polishing. The polished surfaces were etched using 2% nital to reveal the microstructure. Microstructural observations were carried out using scanning electron microscopy (SEM, TESCAN Mira LMS, Shanghai, China) operated at an accelerating voltage of 15 kV and a working distance of approximately 10 mm. Backscattered electron (BSE) imaging was employed to enhance compositional contrast between the oxide scale and the steel substrate. Elemental distributions across the oxide/steel interface were analyzed using energy-dispersive X-ray spectroscopy (EDS) and electron probe microanalysis (EPMA) performed using a JEOL JXA-8100 (JEOL, Tokyo, Japan) electron probe microanalyzer. Particular attention was paid to the spatial distribution and enrichment behavior of Cu and Sn, as well as the influence of Si on the interfacial microstructure and oxidation morphology. Site-specific TEM specimens were prepared from the oxide/steel interfacial region using a focused ion beam (FIB) system (Thermo Scientific Helios 5 UC, Waltham, MA, USA) employing a standard lift-out procedure. Final thinning of the TEM lamellae was performed at low ion-beam energies to minimize Ga^+^ ion damage. Transmission electron microscopy analyses were conducted using a JEOL JEM-F200 (JEOL, Tokyo, Japan) operated at an accelerating voltage of 200 kV. Both conventional TEM imaging and scanning transmission electron microscopy (STEM) modes were employed to characterize the microstructure of the oxide scale and the oxide/steel interface. High-angle annular dark-field (HAADF) STEM imaging was used to enhance compositional contrast. Selected area electron diffraction (SAED) patterns were collected to identify the crystallographic structures of oxide phases and interfacial features. Nanoscale elemental distributions were analyzed using an EDS detector integrated with the TEM.

## 3. Results and Discussions

### 3.1. Oxide Scale Morphology and Interfacial Enrichment of Cu-Sn During High-Temperature Oxidation

High-temperature oxidation experiments were conducted on low-carbon steels containing 0.3 wt.% Cu with 0.05 wt.% Sn to elucidate the coupling effects on oxide scale formation and interfacial enrichment. Samples were oxidized in air at temperatures ranging from 900 °C to 1200 °C for 30 min, simulating thermal histories relevant to hot rolling processes. SEM-EDS analysis revealed distinct differences in oxide scale morphology and elemental distribution at the oxide/matrix interface.

**Figure 2:**
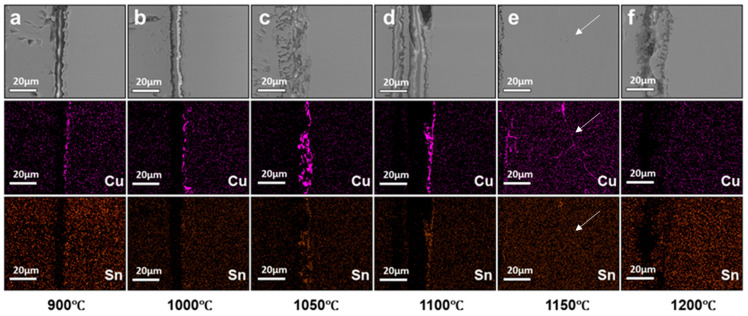
SEM-EDS images of the oxide/steel interfaces of 0.3 wt.% Cu–0.05 wt.% Sn steel after 30 min oxidation at 900–1200 °C, (**a**) 900 °C, (**b**) 1000 °C, (**c**) 1050 °C, (**d**) 1100 °C, (**e**) 1150 °C, and (**f**) 1200 °C. Cu–Sn co-enrichment intensifies with increasing temperature, with liquid phase penetration and interfacial cracking evident at 1150 °C.

For the 0.3Cu-0.05Sn steel, EDS mapping revealed pronounced co-enrichment of Cu and Sn at the oxide/steel interface across a broad temperature range of 900–1150 °C (Figure 2). This coupled distribution is primarily attributed to the presence of Sn, which lowers the melting point of the Cu-rich liquid phase (potentially as low as ~800 °C, based on phase diagram considerations and experimental observations), thereby facilitating enhanced Cu migration and accumulation at the interface even at lower temperatures compared to Sn-free conditions [9,13]. As oxidation temperature increases, the extent of Cu enrichment intensifies, highlighting temperature’s critical role in promoting elemental redistribution [10,14]. At 1050 °C, alongside substantial Cu accumulation, minor Sn enrichment becomes evident, indicating Sn’s tendency to migrate toward the interface under high-temperature exposure (Figure 2c). In contrast to the Sn-containing steel, previous studies on Sn-free steels with similar Cu contents (e.g., 0.05–0.30 wt.% Cu) have shown that Cu enrichment at the oxide/steel interface during high-temperature oxidation is more limited, typically occurring within a narrower critical temperature window of approximately 1100–1150 °C. Further elevation to 1150 °C results in the formation of a Cu-Sn liquid phase that penetrates along grain boundaries, inducing deep cracks exceeding 200 μm and contributing to surface degradation (Figure 2e). The literature confirms that Sn exerts a significant influence on Cu enrichment by co-segregating at the interface, reducing the liquid phase’s melting point, and exacerbating liquid metal embrittlement, which aligns with the observed expansion of the enrichment temperature window and increased susceptibility to hot shortness [15,16]. The oxide scale exhibits a layered structure with outer Fe_2_O_3_, intermediate Fe_3_O_4_, and inner FeO layers, but displays heightened porosity and interfacial irregularity due to the cyclic damage from liquid phase infiltration. At 1200 °C, reverse diffusion of elements back into the matrix predominates over enrichment rates, effectively suppressing interfacial accumulation and yielding a more uniform oxide morphology (Figure 2f). Collectively, these results demonstrate that Sn plays a decisive role in promoting Cu enrichment at the oxide/steel interface, broadening the effective temperature window for enrichment, and influencing both oxide scale integrity and susceptibility to hot shortness. The coupled Cu–Sn behavior underscores the importance of carefully controlling residual elements during high-temperature processing to mitigate interfacial degradation.

**Figure 3:**
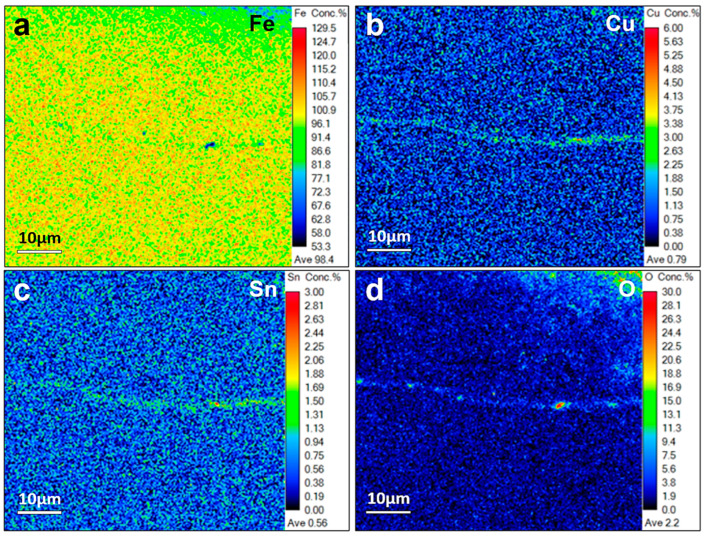
EPMA elemental maps illustrating the “oxidation–penetration” cycle in 0.3 wt.% Cu–0.05 wt.% Sn steel. Cu–Sn liquid penetrates along oxidized grain boundaries. Panels show the distribution of (**a**) Fe, (**b**) Cu, (**c**) Sn, and (**d**) O.

Further electron probe microanalysis (EPMA) characterization revealed (Figure 3) that the Cu-Sn liquid phase penetrates along grain boundaries, leading to grain boundary weakening and the formation of open cracks. Additionally, iron oxides were observed within these cracks, as illustrated in Figure 3d. From a mechanistic perspective, the presence of Sn significantly promotes the infiltration of the liquid phase and the widening of grain boundaries [16]. As the iron oxidation process progresses, the Cu-rich phase gradually thickens, with increasing amounts of oxygen dissolving into the liquid Cu. According to the selective oxidation mechanism, O_2_ reacts with iron at the grain boundaries, resulting in grain boundary oxidation; consequently, iron oxides are detected within the matrix cracks, which coarsen the grain boundary structure and reduce its strength [17]. This weakened grain boundary creates favorable conditions for further penetration by the Cu-Sn liquid phase. As the Cu-Sn liquid continues to diffuse inward along the grain boundary channels, insufficient liquid supply leads to the formation of open cracks near the oxide/matrix interface. These cracks provide diffusion pathways for O, causing further oxidation at the crack sites and the formation of iron oxides [18]. However, as shown in Figure 2f, at 1200 °C, neither cracks nor significant Cu enrichment were observed in the samples. This is primarily due to the excessively high temperature causing the diffusion rates of Cu and Sn to far exceed their enrichment rates, enabling Cu and Sn to reverse-diffuse from the interface back into the steel matrix, thereby inhibiting interfacial accumulation and crack formation. This phenomenon indicates that temperature exerts a dual influence on the diffusion and enrichment behavior of Cu and Sn, where excessively high temperatures may conversely suppress interfacial enrichment.

**Figure 4:**
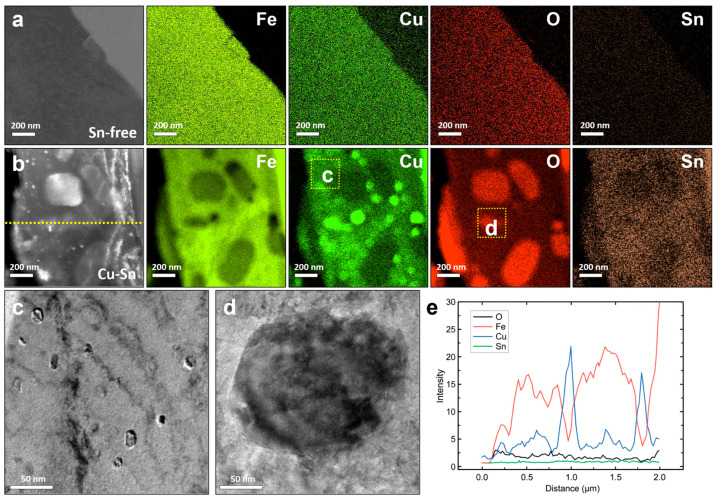
Morphology and elemental distributions at the oxide scale/steel interface for the Sn-free and Cu–Sn steels. (**a**) HAADF-STEM image of the Sn-free steel together with corresponding EDS elemental maps of Fe, Cu, O, and Sn. (**b**) HAADF-STEM image of the Cu–Sn steel and the corresponding EDS maps of Fe, Cu, O, and Sn. (**c**) Bright-field TEM image of a Cu-rich particle magnified from the region indicated by the yellow dashed box in the Cu map in (**b**). (**d**) Bright-field TEM image of a Cu–O-rich particle, taken from the region marked by the yellow dashed box in the O map in (**b**). (**e**) EDS line-scan profiles along the yellow dashed line in (**b**); the intensities are normalized to facilitate comparison between elements.

To gain deeper insight into the microscopic mechanisms by which Sn affects Cu enrichment at the oxide/steel interface, comparative TEM experiments were conducted on Sn-free and Sn-containing steels (Figure 4). After oxidation at 1150 °C for 30 min in ambient air, representing peak temperatures during hot rolling, TEM lamellae were prepared from the oxide/steel interface using FIB milling. STEM-EDS analysis was then performed to characterize the interfacial microstructure and elemental distributions. For the Sn-free steel, the HAADF-STEM image and corresponding EDS elemental maps (Figure 4a) reveal relatively uniform distributions of Fe, Cu, O, and Sn across the oxide/steel interface. No distinct Cu-rich particles or localized segregation are observed, indicating that Cu remains largely dissolved in the Fe matrix under these oxidation conditions. This behavior is consistent with classical selective oxidation models, where Fe preferentially oxidizes while Cu diffusion alone is insufficient to induce phase separation in the absence of interacting residual elements. In contrast, the Sn-containing steel shows clear evidence of Cu redistribution near the interface. As shown in the HAADF-STEM image and elemental maps (Figure 4b), localized Cu enrichment appears in the matrix adjacent to the oxide scale. TEM observations reveal discrete Cu-rich particles in this region (Figure 4c), indicating that the presence of Sn promotes the segregation and clustering of Cu during high-temperature oxidation. In addition, oxygen-containing particles are detected in the matrix of the Sn-containing sample (Figure 4d), suggesting the formation of oxide inclusions associated with the oxidation–penetration process. These O-rich particles are typically surrounded by fine Cu-rich regions, implying that Cu segregation occurs preferentially around oxidized regions in the matrix. The presence of Sn is therefore inferred to significantly reduce the effective solubility of Cu in austenite at the oxide/steel interface, promoting Cu precipitation and facilitating the formation of Cu-rich phases. Thermodynamically, the Cu–Sn system exhibits low melting eutectics, with liquid formation possible below ~800 °C, which can promote liquid phase formation during high-temperature oxidation. Such liquid phases are known to penetrate grain boundaries and weaken interfacial cohesion, thereby increasing susceptibility to hot shortness during hot processing.

Interestingly, despite the Cu enrichment observed in the Sn-containing sample, no obvious Sn segregation is detected within the Cu-rich particles during TEM analysis, with Sn remaining relatively diffuse in the matrix. This difference compared with SEM-scale observations may arise from the faster grain boundary diffusion of Sn compared with Cu. At high temperatures, Sn can rapidly redistribute along grain boundaries and the oxide interface, preventing strong local accumulation while still influencing the thermodynamic stability of Cu in the surrounding matrix. Further insight into interfacial chemistry is provided by the EDS line-scan profiles taken along the dashed line in Figure 4b. As shown in Figure 4e, the normalized intensity profiles reveal pronounced fluctuations in Cu concentration near the interface, while Fe and O display complementary variations corresponding to the oxide/steel transition region. These results provide direct nanoscale evidence that Sn significantly modifies the redistribution behavior of Cu during oxidation. Overall, the comparative TEM observations demonstrate that the addition of Sn promotes localized Cu segregation and the formation of Cu-rich particles near the oxide/steel interface, whereas such features are absent in the Sn-free steel. This difference highlights the important role of Sn in altering Cu redistribution behavior during high-temperature oxidation. By promoting Cu segregation and potentially facilitating liquid phase formation, Sn increases the likelihood of interfacial instability and crack initiation during hot processing. These findings are consistent with previous studies showing that Sn exacerbates Cu-induced hot shortness through both thermodynamic effects, such as lowering eutectic temperatures, and kinetic effects, including enhanced grain boundary diffusion.

The kinetic and thermodynamic contributions of Sn were probed at the nanoscale. Sn’s rapid grain boundary diffusion (estimated ~10^−9^ cm^2^/s at 1100 °C) facilitates Cu precipitation by reducing local solubility in austenite (~20–30% decrease), leading to metastable Cu-Sn clusters that melt and infiltrate boundaries [19]. This infiltration weakens interatomic bonds, as Sn-Sn interactions (~150 kJ/mol) are significantly weaker than Fe-Fe interactions (~400 kJ/mol), promoting capillary-driven penetration. The resulting “oxidation–penetration” cycle—where liquid exposure of fresh matrix drives further oxidation and enrichment—was quantified, with penetration depths correlating to Sn-induced liquid volume.

These mechanistic insights extend beyond morphology, explaining why Sn exacerbates Cu hot shortness more than isolated Cu effects. The lowered eutectic (~800 °C) and enhanced kinetics overlap with hot rolling windows (1000–1200 °C), necessitating Sn control in recycling (<0.02 wt.% threshold for minimal impact). At 1200 °C, Sn’s influence wanes due to dominant reverse diffusion, suggesting thermal optimization as a partial countermeasure. This sets the stage for examining Cu content variations in Sn-present systems, where thresholds for detrimental enrichment are further lowered.

### 3.2. Regulation Mechanism of Si on Cu–Sn Enrichment

**Figure 5:**
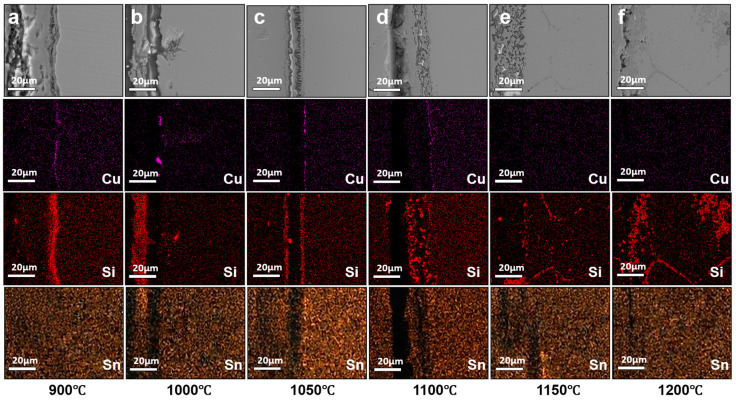
SEM-EDS images of the oxide/steel interfaces of 0.3Cu–0.05Sn-0.4Si steel after 30 min oxidation at 900–1200 °C, (**a**) 900 °C, (**b**) 1000 °C, (**c**) 1050 °C, (**d**) 1100 °C, (**e**) 1150 °C, and (**f**) 1200 °C.

To mitigate the adverse Cu–Sn interfacial enrichment observed in the previous sections, the regulatory effect of Si addition was systematically investigated. Low-carbon steels containing 0.3 wt.% Cu and 0.05 wt.% Sn were alloyed with 0.4 wt.% Si and subjected to high-temperature oxidation at 900–1200 °C for 30 min in air, simulating the thermal conditions experienced during hot rolling to enable direct comparison with the Si-free samples in Figure 2. SEM–EDS and BSE characterization were employed to examine the morphology and elemental distributions at the oxide/steel interface (Figure 5). For comparison, the Si-free steel (discussed in Figure 2) exhibits extensive Cu-rich liquid phases along the oxide/matrix interface. These phases form a continuous interfacial layer and penetrate deeply along grain boundaries (>200 μm) due to Cu–Sn coupling, which lowers the melting temperature of the Cu-rich phase. In contrast, the addition of 0.4 wt.% Si significantly alters the interfacial enrichment behavior. At intermediate temperatures of 900–1100 °C (Figure 5a–d), Cu and Sn enrichment is still detectable but is substantially reduced compared with the Si-free condition. Instead of forming continuous interfacial layers, the Cu-rich phases appear as discrete and isolated particles. This transition indicates that Si interferes with the accumulation and co-segregation of Cu and Sn at the interface. The suppression effect is attributed to Si-induced internal oxidation, which competes with external scale growth and partially redirects residual elements away from the oxide/steel interface. Nevertheless, the persistence of scattered Cu–Sn enriched regions suggests that 0.4 wt.% Si cannot completely eliminate the strong coupling effect when both Cu and Sn content are relatively high. As the oxidation temperature increases to 1150 °C (Figure 5e), Cu–Sn enrichment at the interface is largely suppressed. EDS maps show minimal co-localization of Cu and Sn, indicating that Si becomes more effective at higher temperatures. This enhanced regulation is closely associated with the formation of Fe_2_SiO_4_, which has a melting point of approximately 1177 °C. The presence of this phase provides alternative transport pathways for residual elements, allowing Cu and Sn to migrate into the oxide scale through pores and grain boundaries rather than accumulating at the metal interface. However, the images also reveal significant Fe_2_SiO_4_ penetration along grain boundaries, reaching depths greater than 100 μm. Such penetration may introduce secondary defects or reduce oxide scale adhesion, potentially affecting surface quality during subsequent processing. At 1200 °C (Figure 5f), the oxide/steel interface remains largely free of Cu–Sn enrichment. The higher temperature enhances reverse diffusion of Cu and Sn into the steel matrix, further reducing interfacial accumulation. Meanwhile, the oxide scale becomes more porous, which facilitates the redistribution of residual elements mediated by Fe_2_SiO_4_ phases. Despite the effective suppression of enrichment, the deeper penetration of oxide phases highlights a temperature-dependent trade-off in the regulatory role of Si.

**Figure 6:**
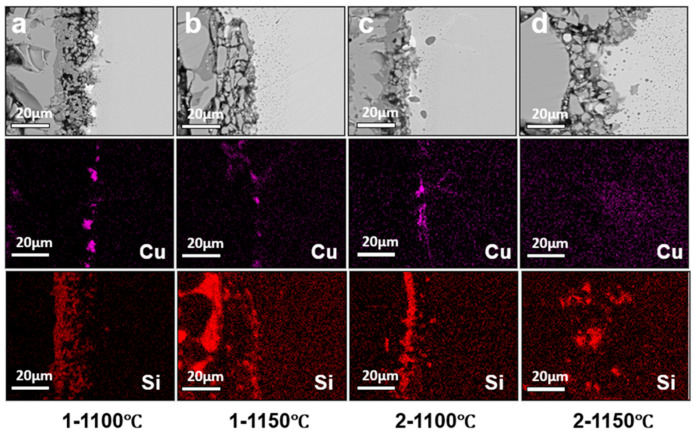
SEM–EDS images of the oxide/steel interfaces of (1) 0.3 Cu–0.05 Sn–0.4 Si and (2) 0.2 Cu–0.05 Sn–0.4 Si steels after 30 min oxidation at temperatures where interfacial enrichment is most pronounced. (**a**,**b**) Sample 1 oxidized at (**a**) 1100 °C and (**b**) 1150 °C; (**c**,**d**) Sample 2 oxidized at (**c**) 1100 °C and (**d**) 1150 °C.

To further explore the compositional sensitivity of Si’s regulatory effect, considering typical residual element levels in industrial recycled steels, we reduced the Cu content while maintaining Sn at 0.05 wt.% and Si at 0.4 wt.%, examining two samples: Sample 1 (0.3 wt.% Cu–0.02 wt.% Sn–0.4 wt.% Si) and Sample 2 (0.2 wt.% Cu–0.02 wt.% Sn–0.4 wt.% Si). BSE and EDS analyses were performed on the oxide/steel interfaces after oxidation at 1100 °C and 1150 °C for 30 min; these are temperatures at which interfacial enrichment is most pronounced in Si-free conditions. For Sample 1 at 1100 °C (Figure 6a), substantial Cu-rich phases are evident at the interface, exhibiting continuous distribution and pronounced penetration along grain boundaries into the matrix, consistent with the partial suppression observed in Figure 5d. This indicates that at this temperature, the Cu–Sn coupling still overwhelms Si’s regulatory mechanisms to some extent, likely due to there being insufficient internal oxidation kinetics to fully redirect Cu migration. At 1150 °C (Figure 6b), however, Cu enrichment diminishes markedly, with liquid Fe_2_SiO_4_ phases observed at the interface and within the oxide layer, surrounded by fine Cu-rich particles. This corroborates the findings in Figure 5e, demonstrating that Si facilitates the formation of low-melting Fe_2_SiO_4_ liquid phases (~1177 °C melting point), which act as carriers for Cu-rich species. Thermodynamically, Fe_2_SiO_4_’s stability and wettability enable it to exploit oxide grain boundaries and pores as diffusion channels, effectively “transporting” Cu away from the interface and preventing sustained accumulation or liquid phase formation [20]. Kinetically, this process is enhanced at 1150 °C, where elevated temperatures accelerate Si diffusion and internal oxidation, reducing the oxygen flux at the interface and weakening the driving force for selective Fe oxidation that typically rejects Cu and Sn [21].

Comparative analysis of Sample 2 (with reduced Cu at 0.2 wt.%) under identical conditions reveals notable differences, highlighting a compositional threshold effect. At 1100 °C (Figure 6c), Cu enrichment features are broadly similar to those of Sample 1, with continuous interfacial layers and grain boundary penetration, suggesting that lower Cu alone does not fully mitigate Sn’s promotional role in enrichment at this temperature. However, at 1150 °C (Figure 6d), no Cu enrichment is observed at the interface, representing a complete suppression of Cu–Sn coupling. This stark contrast implies that when Cu levels drop to 0.2 wt.%, 0.4 wt.% Si can fully inhibit Sn-induced Cu accumulation, possibly because the reduced Cu concentration falls below a critical solubility threshold in austenite, diminishing the thermodynamic potential for phase separation. From an industrial perspective, this finding is significant for scrap-tolerant steel production: recycled steels often contain variable Cu (0.1–0.3 wt.%) from contaminants like wiring, and Sn (up to 0.05 wt.%) from coatings. The results suggest that targeted Si alloying (around 0.4 wt.%) could enable higher scrap utilization by tailoring compositions to exploit temperature windows (e.g., >1100 °C) where Fe_2_SiO_4_-mediated transport dominates, minimizing hot shortness risks without excessive Si additions that might impair ductility or weldability. Nonetheless, further studies are warranted to quantify penetration depths and assess long-term impacts on mechanical properties, as excessive Fe_2_SiO_4_ infiltration could lead to subsurface defects in multi-pass rolling. Overall, these results demonstrate that Si addition regulates Cu–Sn enrichment through a combined thermodynamic and kinetic mechanism. Thermodynamically, Si promotes the formation of Fe_2_SiO_4_ phases that can encapsulate or transport Cu–Sn species, thereby reducing their accumulation at the oxide/steel interface. Kinetically, Si favors internal oxidation and lowers the overall oxidation rate, which decreases the driving force for residual element enrichment. However, the effectiveness of this regulation depends strongly on temperature and composition. At lower temperatures, the strong coupling between Cu and Sn still results in localized enrichment, whereas at higher temperatures the formation and penetration of Fe_2_SiO_4_ phases become dominant. Therefore, optimizing Si content is essential to balance the suppression of Cu–Sn enrichment with the potential risks associated with oxide penetration and surface degradation in recycled steels.

**Figure 7:**
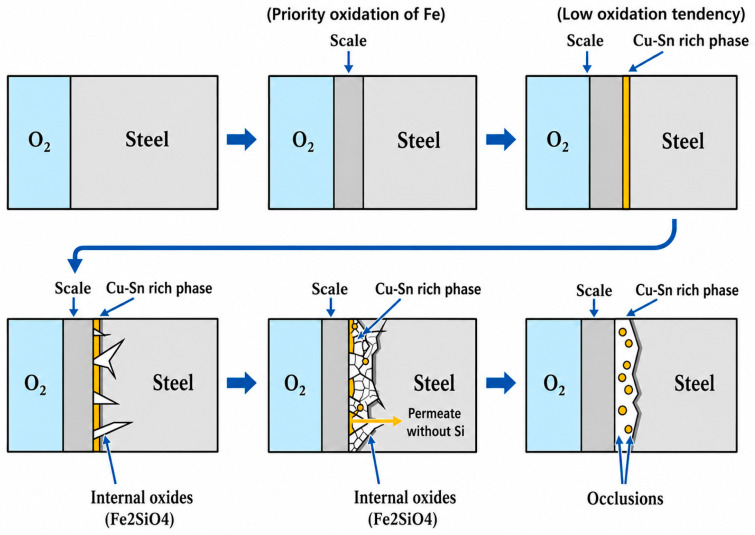
Schematic illustration of the formation mechanism of the irregular morphology at the oxide/steel interface during high-temperature oxidation, divided into three stages: initial selective oxidation with Cu-Sn coupled enrichment, internal oxide precipitation promoting localized Cu-Sn accumulation, and interface intermixing incorporating Fe internal oxides and Cu-Sn phases, regulated by Si through preferential Fe_2_SiO_4_ formation and occlusion into the oxide scale.

The formation mechanism of the irregular morphology at the oxide/steel interface aligns closely with the model proposed by Fukagawa et al. [22], and can be delineated into three primary stages, as schematically illustrated in Figure 7, incorporating the coupled effects of Cu and Sn alongside the regulatory influence of Si. In the initial selective oxidation stage, Fe undergoes preferential oxidation upon exposure to O_2_, resulting in the rejection and gradual accumulation of Cu at the oxide/steel interface. This process is amplified by the presence of Sn, which co-segregates with Cu, reducing the melting point of the Cu-rich phase (potentially to ~800 °C) and facilitating the early formation of a low-melting Cu–Sn liquid film. The diffusion coefficient of Fe in Fe particles (2.3–3.5 × 10^−10^ cm^2^/s) is approximately three orders of magnitude lower than in FeO (2.86 × 10^−7^ cm^2^/s), thereby hindering Fe replenishment and intensifying Cu–Sn enrichment at the interface. During the internal oxide precipitation stage, internal oxides of Fe begin to nucleate and grow beneath the external scale, creating localized regions where Cu–Sn phases preferentially accumulate around these oxides. The synergistic interaction between Cu and Sn promotes enhanced grain boundary diffusion and liquid phase infiltration, leading to intergranular penetration and the initiation of the “oxidation–penetration” cycle, as evidenced by nanoscale TEM observations (Figure 4). This cycle exacerbates surface degradation by widening grain boundaries and fostering crack formation. In the interface intermixing stage, continued non-uniform oxidation leads to the incorporation of Fe internal oxides and localized Cu–Sn enriched zones into the oxide/steel interface, resulting in a convoluted and irregular morphology. The addition of Si plays a pivotal regulatory role here, owing to its higher oxygen affinity compared to Fe. Si promotes the preferential formation of internal SiO_2_ or Fe_2_SiO_4_ oxides at specific depths, which impede the advancement of the oxidation front. This creates a competitive dynamic between internal oxidation and frontal progression, ultimately entangling the steel matrix, FeO, and Fe_2_SiO_4_ phases into an irregular interface structure. Consequently, Cu–Sn rich phases are occluded and redistributed into the oxide scale via pores and grain boundaries, suppressing their interfacial accumulation and mitigating liquid penetration, particularly at elevated temperatures (>1100 °C). This Si-mediated mechanism not only limits the detrimental coupling of Cu and Sn but also enhances oxide scale integrity, offering a practical approach for managing residual elements in steels derived from scrap-containing charges.

## 4. Conclusions

In summary, this study has systematically elucidated the coupled enrichment behavior of Cu and Sn at the oxide/steel interface in recycled steels during high-temperature oxidation, revealing the synergistic mechanisms that exacerbate surface degradation and hot shortness. The presence of Sn significantly intensifies Cu accumulation by lowering the melting point of Cu-rich phases, promoting the formation of liquid films, and facilitating intergranular penetration through an “oxidation–penetration” cycle, which expands the critical temperature window for enrichment and induces severe cracking. Microscale characterizations confirmed that Sn reduces Cu solubility in austenite, leading to localized precipitation and enhanced kinetic effects via grain boundary diffusion.

The addition of Si emerges as an effective regulatory strategy to mitigate these detrimental effects. By promoting internal oxidation and the formation of Fe_2_SiO_4_ phases, Si alters interfacial diffusion pathways, redirects Cu and Sn away from the oxide/steel interface, and suppresses liquid phase infiltration, particularly at elevated temperatures (>1100 °C). However, the efficacy of Si regulation exhibits compositional and thermal dependencies, with optimal suppression observed at lower Cu contents (e.g., ≤0.2 wt.%) and a trade-off involving potential oxide penetration at higher temperatures.

These findings provide critical insights into managing residual elements in scrap-based steel production, enabling enhanced recyclability while maintaining surface quality and hot workability. Future research should focus on quantitative modeling of Si-mediated transport kinetics and multi-element interactions to optimize alloy designs for industrial applications.

## Figures and Tables

**Table 1:** Experimental steel measured chemical composition (wt.%).

No.	C	Mn	Cu	Sn	Si	Fe
Cu	0.064	0.47	0.289	0	0	Bal.
Cu-Sn	0.067	0.49	0.298	0.048	0	Bal.
Cu-Sn-Si	0.066	0.48	0.293	0.049	0.39	Bal.
Cu-Sn-Si 1	0.066	0.48	0.293	0.019	0.39	Bal.
Cu-Sn-Si 2	0.065	0.48	0.196	0.018	0.39	Bal.

## Data Availability

The original contributions presented in this study are included in the article. Further inquiries can be directed to the corresponding authors.
